# Current Practice and Expert Perspectives on Cultural Adaptations of Digital Health Interventions: Qualitative Study

**DOI:** 10.2196/59965

**Published:** 2025-07-18

**Authors:** Vasileios Nittas, Sarah J Chavez, Paola Daniore

**Affiliations:** 1 Department of Behavioral and Social Sciences, Brown University, 121 South Main Street, Providence, RI, 02912, United States, 1 6944624408; 2 Brown University Center for Alcohol and Addiction Studies, Brown University, Providence, RI, United States; 3 Institute for Implementation Science in Health Care, University of Zurich, Zurich, Switzerland

**Keywords:** cultural adaptation, digital health, health equity, intervention, interventions, practice, practices, expert, experts, perspective, perspectives, experience, experiences, qualitative study, interview, interviews, thematic analysis, web-based, mobile health, mHealth, cultural tailoring, digital health intervention

## Abstract

**Background:**

Some people are less likely to benefit from digital health interventions (DHIs) than others. Culture, along with other factors, contributes to these differences. DHIs that do not address a population’s cultural norms or concerns are likely to be less effective. One way to create culturally sensitive DHIs is through cultural adaptations. Yet, there is currently little evidence-based guidance on when and how to adapt DHIs.

**Objective:**

We aimed to capture the experiences of experts to understand the (1) current practices, (2) challenges, and (3) recommendations around culturally adapting DHIs.

**Methods:**

We conducted semistructured interviews (n=15) via Zoom (Zoom Video Communications, Inc) between May and August 2023, with academic experts who have previously undertaken cultural adaptations of DHIs. Experts were identified through publications and snowball sampling. We used a thematic analytical approach, beginning with a preliminary deductive codebook and then following a three-stage analysis. All transcripts were coded with the MAXQDA (VERBI Software GmbH) software. Codes were reviewed, and similar or related codes were categorized into broader themes, consolidating one or multiple codes into a single topic.

**Results:**

Our analysis produced 30 codes, which were categorized into (1) defining culture, (2) justifying the adaptation, (3) choosing the adaptation elements, (4) implementing the adaptation, (5) understanding the challenges, and (6) recommendations. Based on their experiences, experts recommended that (1) the adaptation team is multiprofessional, digitally competent, and culturally sensitive; (2) DHI users and (3) all other relevant stakeholders are continuously involved; and (4) the adaptations incorporate evaluations and knowledge exchange. They further emphasized that culturally adapted DHIs must be understandable, relatable, appealing, and easy to adhere to, ensuring that health technology and content reflect the target population’s lived experiences, sociodemographic characteristics, and digital literacy. When asked which elements of cultural DHI adaptations, the most common responses were language, lived experience, and technology. Responses revealed five common DHI-relevant challenges, including (1) technology, (2) uncertainty, (3) user involvement, (4) communication, and (5) evaluation and sustainability.

**Conclusions:**

The cultural adaptation of DHIs was described as an iterative, often unstructured, and resource-intensive process that requires careful justification and a solid understanding of the culture and the specific cultural group for which it is implemented. Our interviews confirmed the absence of technology-specific frameworks to guide the cultural adaptations of DHIs. Based on our findings, such a framework should guide the choice of the correct definition of culture and the criteria for assessing the need to adapt. It should also offer tools to drive stakeholder engagement, prioritize adaptation elements, and address common challenges.

## Introduction

Digital health interventions (DHIs) use information and communication technologies (ICTs) to prevent, monitor, manage, and treat health conditions [1]. DHIs are often used to support individuals in developing healthy habits (eg, smoking cessation and healthy eating) and improve outcomes for those living with chronic conditions [1]. A key feature of DHIs is that they can provide access to health information and treatments without the need for traditional health care intermediaries such as insurance companies and clinicians [1,2]. DHIs are spearheaded by web and mobile apps, social networking platforms, and self-monitoring devices like fitness trackers, sleep monitors, and wearables (electronic health devices work on the body, such as smartwatches) [2]. Recent systematic reviews indicate that DHIs can be effective and cost-effective in reducing risk and enhancing health outcomes across a variety of conditions, such as diabetes, cardiovascular health, mental health, and addictions [3-5].

Despite increasing access to the internet, mobile devices, and other technologies, medically underserved populations are less likely to benefit from DHIs than others [6-9]. The term “medically underserved” describes those (eg, racial or ethnic minorities) who have limited access to primary health care, primarily due to sociocultural, economic, and geographic vulnerabilities [10]. Two recent systematic reviews on DHIs for physical activity and other weight-related behaviors have demonstrated that the use and effectiveness of these interventions vary across several characteristics, including race, ethnicity, and socioeconomic status [11,12]. Other studies found that African American and Hispanic individuals are less likely to engage with DHIs than their White counterparts [13,14]. Together with other factors, culture has been shown to play a role in these differences because most DHIs are most often designed for dominant cultural groups, limiting their appropriateness for marginalized populations. DHIs that do not address a population’s cultural norms or concerns, thus, are not culturally sensitive, are likely to be less accepted by that population, and therefore, underused or engaged with [10,13,15]. Although culture can influence how people use and engage with a DHI, very few DHIs are designed to be culturally sensitive [8,15]. This often results in DHIs that inherently exclude certain population groups, further exacerbating the digital and health inequities [8]. Although no single definition of culture exists, Castro et al [16] defined it as a group of people’s shared perspectives and practices.

Ensuring that DHIs address the sociocultural norms of a population is challenging. One way to create culturally sensitive DHIs is through adaptations. Cultural adaptations can be defined as interventions that tailor existing, often evidence-based technologies to align with a specific group’s cultural identity, including cultural norms, beliefs, values, and practices [16]. In our previous work, we argued that cultural adaptations may increase DHIs’ reach, engagement, and outcomes for medically underserved subgroups [17]. However, we emphasized that there is currently little evidence-based guidance on when and how to adapt DHIs [17]. The broader literature on cultural adaptations offers various models designed to guide the adaptation process for evidence-based health care interventions [18,19]. These models specify various steps in the cultural adaptation process, such as (1) assessing the evidence base, (2) creating a preliminary adaptation design, (3) testing preliminary adaptations, and (4) refining them into final adaptations. While many of these models offer broad guidance applicable to the cultural adaptation of DHIs, they do not sufficiently recognize and tackle the unique challenges associated with their use of technology. For example, creating a preliminary adaptation design of a mental health intervention will differ drastically when the intervention is delivered physically compared to it being delivered through a mobile phone app that might require expensive and time-consuming software changes. Nevertheless, there have been many noteworthy attempts to adapt DHIs described in the literature to date, culturally, generating essential knowledge and experience that has the potential to contribute to the development of future guidance [20,21].

In this study, we interviewed researchers who have conducted cultural adaptations of DHIs. Through these interviews, we aimed to capture current practices, challenges, gaps, and recommendations for future adaptations. We specifically asked researchers about (1) why and how they adapted their DHIs and defined culture, (2) how they implemented those adaptations, (3) which elements they chose for adaptation and the criteria for their selection, (4) the challenges they faced during adaptation, and (5) their recommendations for future cultural adaptations of DHIs. We hoped that understanding these experiences might help us identify general principles that could be used to develop broader recommendations to help guide future cultural adaptations of DHIs.

## Methods

### Study Design

We conducted semistructured 45-minute interviews (n=15) with researchers who have previously undertaken cultural adaptations of DHIs. Researchers (hereafter referred to as experts) were invited to participate in this study if they (1) developed and implemented at least one cultural adaptation of a DHI (globally) and (2) either currently hold an academic appointment or have had a past academic appointment.

### Participants and Recruitment

We initially identified eligible experts through academic publications. In March 2023, we searched PubMed and Google Scholar using keywords related to DHIs, culture, and adaptations. We used the following keywords: “digital health,” “digital health intervention,” “mobile health,” “web-based health,” “e-health,” “culture,” “socio-cultural,” “cultural tailoring,” “cultural adaptation,” and “cultural sensitivity.” Relevant publications had to address an actual cultural adaptation of a DHI, irrespective of the health field or population. One reviewer (VN) screened and selected all relevant studies. We screened 138 studies, of which 37 were deemed relevant and appropriate and were used for extracting all corresponding authors’ names and email addresses. Invitations were emailed to participants, where we briefly introduced the project’s aims and participation requirements. In case of nonresponse, we sent reminder emails 2‐3 weeks after initial contact. Those who expressed interest were emailed a bulleted summary of the project and asked to provide their feedback on availability. We identified additional experts through snowball sampling, which involved asking experts at the end of their interview if they knew of other researchers who may be eligible. Inclusion criteria were verified by screening a recommended expert’s publication list to ensure experts had (1) at least one publication specific to the cultural adaptation of a DHI and (2) a past or current academic appointment. We purposively chose interviews over a literature review because the current DHI literature primarily provides superficial descriptions of the adaptation processes. Instead, interviewing experts allows for a deeper and more nuanced understanding of current practice, challenges, and gaps, and provides experience-based recommendations. Interviews were concluded once we reached thematic saturation. We defined thematic saturation as reaching a state where conducting new research does not provide substantially new information or additional conceptual depth but rather a repetition of themes. We stopped interviewing once we felt that a significant amount of information was repeated, which occurred after 13 interviews. We then conducted two additional interviews to confirm that thematic saturation was reached.

### Data Collection

We used a semistructured interview guide developed by the research team and informed by a literature review that we previously conducted (Multimedia Appendix 1) [17]. We used the main topics that emerged in the literature review to develop the interview sections and questions. Interviews were divided into four parts that were specific to DHIs. The first part was an introduction (eg, “tell us about your background and overall experience in cultural adaptations”); the second part focused on the justification of cultural DHI adaptations (eg, what made you decide to adapt?). Furthermore, the third part consisted of questions around adaptation elements (eg, what elements did you adapt and how did you select these elements?), and the fourth part addressed challenges, solutions, and recommendations (eg, what challenges did you face during the adaptation process?”). Based on the first two interviews (pilot), we iteratively adjusted the interview guide. Specifically, the wording of parts three and four was adjusted to be more targeted and more precise, with a series of narrower subquestions.

One interviewer (VN; epidemiologist) with previous experience in qualitative research and interviewing conducted the interviews via Zoom (Zoom Video Communications, Inc) between May and July 2023. Each interview began with a verbal summary of basic information about the study and its objectives. Experts had the opportunity to ask questions. All sessions were recorded and transcribed with Zoom’s transcription software. Additional notes were taken by the interviewer during each interview. Each transcription underwent quality checking and was corrected by one of the research team members (VN). Before analyzing the data, we deidentified all transcripts and removed any references to names, institutions, and project names. Each expert was given a unique identification number linked to their transcripts. A week after transcribing, we deleted all recordings.

### Analysis and Synthesis

We applied an iterative, thematic analytical approach [22]. We initially created a preliminary deductive codebook based on our prior work [17]. Specifically, we used the challenges identified in our review to create initial codes (eg, adaptation justification). We then followed a three-stage analysis to ensure themes were understood and developed thoroughly. First, we read and reread all the interviews to familiarize ourselves with the data. That stage led to inductive modifications of the codebook. Second, all transcripts were coded by VN using the MAXQDA software (VERBI Software GmbH). Third, codes were reviewed, and similar or related codes were categorized into broader themes, consolidating one or more codes into a single unit topic. We then described and narratively synthesized these themes. Following standard practice, a second coder (PD) independently coded about 15% (n=2) of our interviews (PD) to ensure that no critical codes were missed [23]. Finally, we synthesized our data and created themes that represented what most experts were stating. Additionally, we elaborated on emerging findings that warrant further investigation.

### Ethical Considerations

An ethics approval and informed consent were not required as this study does not meet Brown University’s institutional definition of human participant research [24]. An ethics application was submitted and was dismissed as nonhuman participant research. All study data have been anonymized and deidentified. Recordings have been permanently deleted. Each expert received a US $100 voucher.

## Results

### Overview

We invited 34 researchers to participate in our study. A total of 25 (74%) researchers responded to our email, and 15 (44%) researchers agreed to be interviewed. Of those, 11 (70%) researchers were directly identified through publications, and 4 (30%) were through referrals. In total, 13 of the experts were female. Participant characteristics are listed in Table 1.

**Table 1.** Participant characteristics.

ID	Country	Area of expertise	Adapted technology	Target population
1	Brazil	Public health	Mobile app	Pregnant woman
2	United States	Oncology	Web-based decision aid	Latino men
3	Germany	Psychology	Mobile interventions	Low-income setting
4	United Kingdom	Caregiving	eHealth training	Young carers
5	Switzerland	Physical activity	Mobile app	English speakers
6	New Zealand	Public Health	Text-based app	Indigenous
7	Canada	Nursing	Web-based program	Low-income setting
8	United Kingdom	Psychology	eHealth training	Minority carers
9	Sweden	Psychology	Web-based intervention	Minority youth
10	Spain	Caregiving	eHealth training	Informal caregivers
11	Switzerland	Psychology	Mobile app	Migrants
12	Germany	Psychology	Digital intervention	Refugees
13	Germany	Psychology	Mobile app	Refugees
14	Greece	Psychology	Web-based program	Informal caregivers
15	Spain	Public health	Web-based program	Adolescents

Our analysis yielded 30 codes, which we categorized into six broader themes aligned with our interview guide. Table 2 provides a description of all themes and subthemes, which includes: (1) defining culture, (2) justifying the adaptation, (3) choosing the adaptation elements, (4) implementing the adaptation, (5) understanding the challenges, and (6) recommendations. The first theme addresses topics related to all preparatory steps before a cultural adaptation is implemented. Themes 2 to 5 address issues directly related to implementation.

**Table 2.** Definitions of themes and subthemes.

Themes and subthemes	Description
1. Defining culture	Main theme that includes all statements referring to the definition, perception, and conceptualization of culture.
2. Justifying the adaptation	Main theme that includes all statements referring to reasons for culturally adapting DHIs^a^.
2.1. Pragmatic reasons	Subtheme that includes all opportunity-driven reasons to culturally adapt DHIs.
2.2. Equal access	Subtheme that includes reasons to culturally adapt DHIs that refer to equal access to technology.
2.3. Other ethical reasons	Subtheme that includes all other ethical reasons to culturally adapt DHIs.
3. Implementing the adaptation	Main theme that includes all statements referring to implementing cultural adaptations of DHIs.
3.1. The adaptation team	Subtheme that describes the role of adaptation team members in the implementation of adaptations.
3.2. User involvement	Subtheme that describes the role of user involvement in the implementation of adaptations.
3.3. Expert or stakeholder involvement	Subtheme that describes the consulting role of experts or stakeholders in the implementation of adaptations.
4. Choosing the adaptation elements	Main theme that includes all statements referring to selecting and prioritizing what DHI elements to adapt.
4.1. Language	Subtheme that describes language as a critical adaptation element.
4.2. Lived experience	Subtheme that describes lived experience as a critical adaptation element.
4.3. Technology	Subtheme that describes technology as a critical adaptation element.
5. Understanding the challenges	Main theme that includes all statements referring to challenges when adapting DHIs.
5.1. Technology	Subtheme that describes adaptation challenges related to technology and its functions.
5.2. Uncertainty	Subtheme that describes adaptation challenges related to missing knowledge or evidence and uncertainty
5.3. User involvement	Subtheme that describes challenges related to involving and engaging users in the adaptation process.
5.4. Communication	Subtheme that describes challenges related to communication during the adaptation process.
5.5. Evaluation and sustainability	Subtheme that describes challenges related to evaluating and sustainably maintaining an adapted DHI.
6. Recommendations	Main theme that includes all statements referring to expert recommendations for future cultural adaptations of DHIs.

a DHI: digital health intervention.

### Defining Culture

We asked participants how they define culture, primarily in the context of their work. Most describe culture as a complex concept beyond ethnicity and place of origin. Instead, they emphasized (1) community, (2) shared experience, (3) shared values, (4) family, and (5) sociodemographics, as well as (6) shared context and living conditions. Of those experts who provided more details, defining culture (in the context of DHI adaptations) was described as an intuitive and pragmatic process often grounded in common knowledge (eg, what people think they know about culture) or the adaptation team’s expertise of the target culture (eg, the culture of the focal population that a DHI is adapted for). Overall, many experts described culture as an influencer of one’s perception of the world, which includes the perception of health and technology. One expert summarized that as “cultural and context,” and described cultural adaptations as “contextual adaptations.” One expert who has adapted a mobile mental health app for refugees described:

*But what you have to do is you have to do contextual adaptation, you have to understand the context in which the people grew up in which they live now, and in which they will use the app.*

[ID13]

### Justifying the Adaptation

#### Overview

We asked participants about valid reasons to culturally adapt to a DHI, as well as the reasons for their adaptations. Replies led to three subthemes: (1) pragmatic reasons, (2) providing equal access to DHIs for underserved populations, and (3) other ethical reasons.

#### Pragmatic Reasons

Some experts mainly mentioned the lack of guidance specific to cultural adaptations of DHIs. A participant (ID9) who has previously adapted a web-based health intervention for minority youth confirmed: “I don’t think there is much in the existing research that can guide us in that.*”* Therefore, their decisions to adapt were often described as intuitive and common sense–based (eg, they believed they were needed and they made sense), as well as the result of arising opportunities, such as available funding for transferring a DHI to another community. One expert (ID8) who has worked on the adaptation of an eHealth training for young carers in the United Kingdom said: “Our adaptation came about through a recommendation from our funders.”

#### Equal Access

One consistent theme that arose when justifying their adaptations was the provision of equal access. It was described as making digital health resources available to people who are expected to benefit but tend to be medically underserved and would not be reached otherwise. This was described as the case either because the nonadapted DHI is (1) not relevant or appealing, (2) not understandable, (3) offensive, (4) incompatible with prevailing norms and values, and (5) not aligned with the context and lived experiences. An expert who has adapted an eHealth training for minority young carers described:

*There was very, very little in terms of resources for them, support in the caring world [...] there is a lot of evidence south Asian minorities, communities in the UK have a different look at what caring means, and because their family context is quite different, their openness or the way they like to receive the help is quite specific.*

[ID4]

#### Other Ethical Reasons

One expert emphasized the ethical reasons for adaptation, including (1) eliminating Western biases from DHIs before implementing them in more culturally diverse environments and (2) enhancing the visibility of individuals who are otherwise socially excluded groups. One expert said:

*But we knew there wasn’t anything for younger carers of people living with dementia [...] we could do something that at least was going to help increase the visibility of the group.*

[ID4]

Another common theme was the improvement of DHI engagement and adherence, specifically for people who have otherwise previously shown low engagement intervention rates. Here, adaptations were described as synonymous with an elevated user experience, which ensures that people interact with a DHI long enough for it to have an effect. An expert who had previously adapted a mental health app for refugees described:

*So, the ultimate goal for cultural adaptation is really to make sure that the core of the intervention can even take effect. And people do not drop out before they get to the point.*

[ID13]

Two additional adaptation reasons that experts gave were (1) to understand whether a DHI is suitable for implementation across sociocultural settings and (2) to implement a DHI that is initially generic. For instance, a DHI that has been successfully adapted at least once indicates that it is transferable and potentially scalable across sociocultural contexts. Similarly, many DHIs were designed to be adapted (eg, some generic interventions developed by the World Health Organization [WHO]), which was the case for many of our experts. One mental health expert said:

*The intervention in itself is set up to be adapted. So it is developed by the World Health Organization that is recommending that researchers and research groups pick this up and adapt it.*

[ID8]

### Implementing the Adaptation

#### Overview

Experts’ views and experiences on the implementation of the cultural adaptation focused on (1) the adaptation team, (2) user involvement, and (3) expert and stakeholder involvement. The adaptation process of DHIs was described as dynamic, iterative, often unstructured, and trial-and-error-based.

#### The Adaptation Team

Overall, responses revealed two critical characteristics of a good cultural adaptation team. First, a multiprofessional background, which was described as a team with a diverse skill set and professional expertise, including experts in health and technology, such as hardware and software designers. An expert who adapted a mobile health intervention for refugees described:

*So, you need lots of people with very different backgrounds, and you need them to work together efficiently [...] If you want to have a proper user experience, you need a designer. You need people who know something about user interfaces.*

[ID13]

Second, cross-cultural competence, which includes language skills (written and verbal), is another factor in successful DHI adaptations. Experts described that this could be experts (eg, researchers, citizens, or community members) with the same cultural background (eg, lived experience) as the focal population. Cross-cultural competence was described as enhancing cultural credibility, which facilitates the reach and engagement of the target population in the adaptation process. Additional specific roles and expertise requirements were unclear, aside from providing cultural expertise and facilitating communication with the focal point population. Some experts highlighted that cultural adaptation teams often lack these characteristics due to a lack of time and resources.

#### User Involvement

Overall, experts viewed user involvement (of the focal cultural group) as the gold standard approach to culturally adapting DHIs. Good user involvement was described as (1) timely (from the start of the adaptation), (2) iterative and stepwise (allowing for multiple rounds of feedback), and (3) continuous (throughout the adaptation process). Yet it was also mentioned that there are no clear guidelines on how to involve users. An expert (ID12) who worked on the adaptation of the DHI for refugees mentioned:

*The field is new ... there is no blueprint, so you have to make it participatively and try to see what really works within that culture in the sense of what is their understanding.*

[ID12]

Responses highlighted several benefits of user involvement, including (1) a deeper understanding of technology use within a specific culture, (2) capturing lived experiences and comprehending the extent of necessary adaptation, (3) identifying and recognizing diversity within cultural groups (eg, subcultures) and adjusting accordingly, (4) increased chances that the adapted DHI aligns culturally with the target group, (5) a higher likelihood of ongoing use, and (6) fewer errors (eg, adapting something that requires no change). Avoiding mistakes was highlighted as particularly relevant to DHIs by one of our experts, who adapted an eHealth training for informal caregivers, primarily because changing software or hardware is associated with lower cost-effectiveness of the adaptation process. They mentioned:

*Well, advantages, many in terms of that you probably will get a product that is going to be hopefully used [...] you will reduce or limit the amount of mistakes that you will have if you don’t do it. And that is important for cost-effectiveness.*

[ID10]

Another expert highlighted that recruiting must be culturally tailored and supported by relevant community groups and organizations to reach and engage users in the adaptation process.

Responses revealed three methods for user involvement, primarily through interviews and focus groups. The first approach was expert-centered, in which the adaptation team and consulted experts made all initial decisions on which DHI elements must be adapted and how (top-down approach). The second approach included simultaneous expert and user feedback, mainly in the form of interviews and focus groups (hybrid top-down and bottom-up approach). The third approach was user-centered, commonly agreed to be best practice, in which users were continuously involved in the adaptation process (through surveys, interviews, and focus groups), and expert opinions were mainly confirmatory (bottom-up approach). An expert who adapted a text-based app for Indigenous communities in New Zealand said:

*Always start with the people. So, we generally always start with some formative research, focus groups, or other kind of qualitative research with that group you know, kind of representing the target population.*

[ID6]

Overall, this approach was often described as the most resource-intensive.

One expert highlighted that even if the adaptation of the technology is behind (eg, the software has not been adapted yet), it is essential to ensure users are provided with regular input (mock-up versions, examples) to ensure that user feedback is as helpful as possible. Yet, experts highlighted that due to a lack of time and resources, in practice, user involvement often plays a more minor role than it should in cultural adaptations of DHIs. One expert in the cultural adaptation of DHIs in middle and low-income settings mentioned:

*I didn’t have a lot of money. So, it would be nice to include more participants [...]. So, like If I had more time, I definitely would have done a qualitative study to get feedback on the adapted version before conducting the pilot’s study.*

[ID3]

Overall, involving DHI users, the adaptation process was described as iterative.

#### Expert and Stakeholder Involvement

Our participants also addressed the integration of experts and other stakeholders (eg, health care professionals and software designers) into the adaptation process, in addition to user involvement. This was distinguished from having experts or stakeholders within the adaptation team, and it mostly referred to consulting additional ones throughout the adaptation process. An expert said:

*So all the right stakeholders, the funders, the referrers, the service providers in that kind of area that you’re in [...] just try and think of them all up front and then include them in your project, if you can.*

[ID6]

Overall, responses highlighted that experts and stakeholders can provide critical knowledge (eg, technical), facilitate user involvement in the adaptation process (eg, doctors referring patients), and contribute to an adaptation’s long-term success. One expert described the role of experts and stakeholders as supportive, by reducing the overall uncertainty around cultural adaptations.

### Choosing the Adaptation Elements

When asked which elements of cultural DHI require adaptations, the most common responses were: (1) language, (2) lived experience, and (3) technology.

#### Language

First, experts explained that the language of a DHI, including its tone, narrative, and complexity, must be clear and relatable to the focal population. One expert in cultural adaptations for young carers described:

*[...] just the language and not necessarily meaning translation. Just the whole tone of the language, the simplicity, clarity of the language.*

[ID4]

#### Lived Experience

Second, they viewed that adapted DHI content must reflect the lived experience of the focal cultural group, which includes their reality, past and current living conditions, values, beliefs, and preferences. An expert in caregiver DHIs described:

*We then definitely wanted to collect a number of lived experiences that we could then use to create test case scenarios.*

[ID4]

#### Technology

Third, experts noted that culturally sensitive changes in technology and its functions must take into account the focal group’s digital literacy and skills, particularly if the group’s cultural influences play a role. An expert on the adaptation of mobile mental health interventions for refugees said:

*We made sure that it looks and works similar to what people are already using [...]. So people are already familiar with the interfaces and how it works to support those with low tech literacy.*

[ID3]

That ensures that the adapted DHI is easy to adhere to. Experts also mentioned that it is essential to carefully consider the sociodemographic characteristics of the focal population, such as age and gender, as these often interact with one’s cultural background, influencing how health technologies are perceived and used.

When asked how to choose what to adapt, responses varied and did not suggest any specific models or frameworks to help prioritize which DHI elements to adapt, except for those who conducted implementations of generic DHIs, where the developers of the initial intervention provided some adaptation guidance.

### Understanding the Challenges

Responses identified five common DHI-relevant challenges, including (1) technology, (2) uncertainty, (3) user involvement, (4) communication, and (5) evaluation and sustainability.

#### Technology

DHIs were described as more complex to adapt than non-DHIs because changing hardware and software often requires more resources (expertise, time, and money), including higher up-front investments than usually expected or available. An expert described:

*So a couple of months ago, we needed to update one of our digital tools. You swear we were trying to put somebody on the moon. We’re changing something that’s very basic. But it’s going to be $6000. It is taking so much longer. It was just changing a few words.*

[ID7]

Experts also described that if resources are limited, the cultural adaptation risks being insufficient or superficial, which was the case for many of the examples mentioned during the interviews. One expert said:

*We were very limited in budget, so ideally, the adaptation would have been a lot more radical, and it would have had animations.*

[ID4]

Also, two experts emphasized that current health technologies are based on, and therefore biased toward, Western cultures. Adapting these technologies without any influence of these biases and preconceptions was described as a complex yet crucial task.

#### Uncertainty

Uncertainty was implied in most interviews and primarily referred to missing knowledge of the required cultural adaptation. Experts often described not knowing the right balance between adapting a DHI, keeping costs contained, and ensuring that the adapted DHI is effective. An expert in text-based DHIs for Indigenous communities mentioned:

*You can never fully account for everything that you need to in terms of cultural adaptation. So there are always things that you don’t know.*

[ID6]

Adapting too much was described as too costly, leading to DHIs that are too specific, and thus, exclude some people who might otherwise have benefited. Adapting too little was described as being insufficiently targeted, which may result in ineffective adaptations and wasted effort and resources. Many aspects outlined in the previous paragraphs (eg, ensuring that the adaptation team is multiprofessional and culturally competent, involving users, experts, and other stakeholders, and evaluating adapted DHIs) were mentioned as potentially reducing some of that uncertainty. Ultimately, one expert described that uncertainty is inherent and cannot be avoided entirely.

#### User Involvement

Overall, responses highlighted that user involvement in the adaptation process can be challenging, mainly when conducted digitally and remotely (often in DHIs). Experts mentioned that it is difficult to (1) reach people and get them engaged, (2) bring experts and users to collaborate, and (3) secure enough time and money for thorough user involvement. An expert in the adaptation of maternity mobile health apps in Brazil said:

*I tried it, but there are categories of power, and I was less powerful. So, to get these people (experts) together and convince them that this was important was already very difficult. Imagine having the users involved.*

[ID1]

As explained by the experts, these challenges, in addition to limited resources (money and time), often lead to cultural adaptations that have minimal to moderate (eg, small sample sizes and few feedback loops) user involvement, often after the adaptation team and experts have decided on what and how much needs to be adapted.

#### Communication

Experts emphasized that cultural adaptations, especially of DHIs, are a multidisciplinary effort and rely on feedback from various stakeholders across disciplines (eg, technology, medicine, and public health). According to their experience, communication was viewed as a common challenge. This primarily involved communication among IT experts, the rest of the adaptation team (eg, health researchers), and other stakeholders, including users. For example, one expert in informal caregiver DHIs mentioned that translating user and expert feedback in understandable ways for hardware and software designers was described as particularly challenging, saying:

*So, I was in between the target population and the engineering developers of the platform. And it’s very difficult to have a communication with both and try to put them together.*

[ID10]

Maintaining transparency and including team members with interprofessional skills (eg, health and engineering knowledge) were mentioned as potential solutions to communication problems. Yet, some responses indicated that the lack of resources and time does not always allow for that.

#### Evaluation and Sustainability

Experts mentioned that robust evaluations (eg, randomized controlled trials) of how well culturally adapted DHIs perform are rare. Two common themes that arose were (1) the scarcity of additional resources to conduct evaluations and (2) the difficulty of engaging often hard-to-reach individuals in evaluations (eg, in longitudinal studies). Finally, culturally adapted DHIs were described as dynamic and may need to be regularly updated to remain relevant. Our participants described that as a challenge because (1) technology or software changes can be complex and costly, (2) lacking postadaptation funding, and (3) lacking evaluations that prove the initial cultural adaptation was effective. Overall, responses suggested evidence of the effectiveness of cultural adaptations of DHIs is currently lacking, which explains the lack of systematic guidance (eg, DHI-specific models or frameworks). An expert (ID6) in text-based DHIs described:

*[...] they generally don’t even put enough money into maintaining it because they just think once you’ve done a digital program, it’s there. And so then they tend to put funding in for things like upgrades of digital systems.*

[ID6]

### Recommendations

Based on all our findings, we have compiled a list of recommendations for future cultural adaptations of DHIs. All stem from the experiences, mistakes, and lessons learned by the interviewed experts. We summarized all recommendations in Textbox 1.

Textbox 1. **Recommendations for future cultural adaptations of digital health interventions (DHIs).**

- Define the focal culture or cultural group clearly from the start
- Learn from the experience of those who have previously culturally adapted DHIs
- Involve users continuously, adapt only what is relevant to them, and consider their digital literacy
- Think about the DHI design and functionality from the start, and prioritize what users want
- Do not underestimate the money and time needed to adapt technology and expect setbacks
- Stay open-minded and free of assumptions (ie, do not assume what the focal population wants)

The first recommendation highlights the importance of a clear, appropriate definition, chosen well before an adaptation starts. An expert on mobile mental health intervention mentioned:

*You really need to define culture as something trans-cultural, something diverse, and you have different categories that you have to keep in mind.*

[ID11]

The second recommendation addresses current evidence gaps and urges future adaptations to learn from existing knowledge, either through reviews or by talking to people who have adapted DHIs. One expert in the cultural adaptation of DHIs in middle and low-income settings said:

*There are some projects doing cultural adaptations of the digital interventions and like in a in a very systematic way. So, I think it’s good to talk to them to get their experiences.*

[ID3]

The third recommendation emphasizes the need to involve users throughout the adaptation and ensure that all changes to a DHI reflect their needs and preferences. While involving users in the adaptation process, it is important to understand and address their digital support needs. One expert highlighted: *“*But I mean, even involve them at the very, very earlier stage of the research. I think that would be the best thing*.*” Related to that, thinking early about technological changes (eg, changing certain functionalities for better cultural fit) is the fourth recommendation. One mental health expert (ID8) said:

*So thinking about the web build and the functionality from the outset rather than as a second thought. Because, yeah, it’s kind of the cultural adaptation, the content, and like I said that it could work.*

[ID8]

The fifth recommendation urges adaptors to consider the time and money that adaptations require before adapting. One expert (ID13) who has adapted a mobile mental health app for refugees mentioned:

*And then, another thing is, it is really important not to underestimate the money and time it takes to create software.*

[ID13]

The final recommendation refers to adapting without assuming what the focal population wants, but listening carefully and only acting on that. An expert who has adapted an eHealth training for minority young carers described:

*I just think it’s getting rid of your preconceptions and your assumptions about the cultural group that you are targeting.*

[ID4]

## Discussion

### Principal Results

Cultural adaptations are emerging and have the potential to improve DHI access for medically underserved population groups [25]. This work represents one of the first attempts to learn from the experiences of academic experts who have previously culturally adapted DHIs. Our findings outline current practice and underline existing gaps. Overall, the current practice emphasizes culturally competent and multiprofessional adaptation teams that involve IT experts (eg, hardware and software designers), and user, expert, and other stakeholder involvement in the adaptation process. There is also an emphasis on three core adaptation elements, highlighting that a culturally adapted DHI must be understandable (eg, using appropriate language, tone, and narrative), relatable (eg, reflecting lived experience and sociodemographics), and appealing and easy to adhere to (eg, functions, design, and layout that consider digital literacy and preferences). However, many challenges remain, and although the field is emerging, responses have not revealed any systematic ways for the cultural adaptations of DHIs.

### Comparison With Prior Work

Our findings are partially aligned with general adaptation frameworks, such as FRAME (framework for reporting adaptations and modifications to evidence-based interventions) [26]. Our findings, and FRAME, highlight the importance of justifying adaptations, clearly outlining adaptation elements, involving a broad range of stakeholders (from experts to community members), and conducting evaluations [26]. Our findings also align with the 17 cultural adaptation considerations provided by the systematic review of Spanhel et al [27] for internet- and mobile-based mental health interventions [26]. In particular, they confirm the importance of adapting in ways that reflect the lived experience of the target population. That includes, as also identified by Spanhel et al [27], habits, gender norms, religious beliefs, values and traditions, stressors, and socioeconomic factors (eg, education and political context) [27]. Spanhel et al [27] also underlined the importance of language tailoring and visualization, ensuring that text is simple, culture-specific, and accompanied by visual cues [27]. This aligns well with our findings, highlighting that adaptation goes well beyond simple translations. Experts described that language, including its tone, needs to be understandable, providing a relatable and culturally familiar narrative. Our work contributes beyond that and provides insights into implementers’ challenges when adapting digital interventions. Securing enough resources to conduct thorough hardware and software adjustments, reaching and engaging users of the focal cultural group (digitally or remotely) throughout the adaptation process, and ensuring good communication between ICT and non-ICT members of the adaptation team were some of the technology-specific challenges that experts described.

Uncertainty was emphasized as one of the most prominent and cross-cutting adaptation challenges. That means adaptations often start with little knowledge of how much to adapt to ensure DHI effectiveness without wasting time and resources. Uncertainty is not exclusive to DHIs but to cultural adaptations in general [28]. Several well-described challenges might lead to uncertainty. Castro et al [16] describe some of these and include (1) defining culture, (2) segmenting and understanding the population for which the adaptation is conducted, and (3) understanding heterogeneity and subcultures within that population. Although experts provided recommendations to mitigate uncertainty when adapting DHIs (eg, continuously involving the user and expert feedback), the lack of evidence, guidance, frameworks, and systematic practice makes uncertainty inherent. That uncertainty was repeatedly voiced during the interviews and is reflected in our findings overall.

When asked how to choose or prioritize what to adapt, responses varied and did not provide any systematic approach. User-centeredness, although not always possible if resources are lacking or the target population is hard to reach, was highlighted as the best approach for identifying (or prioritizing) which DHI elements are culturally relevant and worth adapting. This aligns with previous work highlighting the value of user-centered adaptations, particularly when adapting DHIs [29]. The acceptance and use of health technologies depend on many factors, such as self-efficacy, prevailing norms and values, and trust [30]. These factors are often related to culture, and therefore, highly relevant to the adaptation process. Without direct feedback from prospective users and members of the focal cultural group, these factors cannot be fully captured. Based on the experience of these experts, engaging users should start early in the adaptation process, be iterative, allow for flexibility, be continuous yet stepwise, and allow for feedback loops. However, there is still little systematic guidance on how to involve DHI users in the cultural adaptation process, especially if conducted digitally or remotely.

### Implications, Current Practice, and Existing Gaps

Our findings highlight the importance of cultural adaptations for reaching and engaging systematically underserved communities. Despite overall agreement on their value, current practice often lags, and the needed support to advance the field has not been prioritized yet. First, there seems to be a lack of dedication to resources to complete well-designed and thorough adaptations, which leads to adapted DHIs that are only partially adapted and not adequately evaluated. This is aligned with the findings of a recent literature review by Naderbagi et al [31], which highlights the lack of rigorous DHI adaptations, arguing that budgets and timelines should facilitate instead of limiting the adaptation process.

Second, the lack of thorough evaluations likely has a negative impact on the field as it limits available evidence on which future DHI adaptations could be built. Existing frameworks, such as the Ecological Validity Framework, the Cultural Accommodation Model, and the circular model of cultural tailoring, offer valuable higher-level guidance on cultural adaptations, yet do not sufficiently cover aspects specific to DHIs, such as technological and digital literacy barriers [32,33]. Recent work, such as the systematic review on the cultural adaptation of internet- and mobile-based interventions for mental disorders by Spanhel et al [27] and the related editorial by Marwaha and Kvedar [25], provides valuable steps toward filling that gap. However, a comprehensive framework focusing on the cultural adaptation of DHIs is still lacking. That feeds an uncertainty on what to prioritize to maintain a balance between available resources and addressing the population’s needs, the adaptations are aimed at. Our findings contribute to closing the practice gaps by (1) highlighting actual practice gaps and DHI-specific challenges, (2) providing examples of commonly agreed-upon good practices, (3) identifying core adaptation elements, and (4) highlighting the need for DHI-specific cultural adaptation frameworks.

### Toward DHI-Specific Cultural Adaptation Frameworks

Our methodological approach does not allow for the development of a DHI-specific cultural adaptation framework because it is a single qualitative study with a limited range of participant perspectives. Developing a robust and transferable framework typically requires insights from multiple studies and across a diverse range of participants, as well as data triangulation [34]. However, our findings contribute toward a future DHI-specific cultural adaptation framework. First, our findings highlight the importance of defining culture and justifying the adaptation early on. A future framework should provide stepwise guidance on how to identify relevant cultural elements in ways that align with a specific DHI and the targeted cultural group, as well as tools to identify cultural nuances beyond ethnicity and place of origin. It should also, as our findings highlight, provide criteria for assessing the need to adapt.

Second, our findings indicate the importance of diverse adaptation teams, as well as user and stakeholder engagement. This is aligned with prior work for nondigital interventions that emphasize the role of community and stakeholder involvement in sustainable and efficient adaptations [35]. A DHI-specific framework should (1) guide the creation of adaptation teams with adequate digital expertise, and (2) provide digital approaches to reach and engage users and other stakeholders. This would complement existing work, which highlights the importance of involving the target group in the adaptation process but offers little guidance on digital ways to do so [27].

Third, our findings present essential elements of adaptation but emphasize that these decisions are context-specific. A future framework should offer support for decision-making related to prioritizing which elements need adaptation, including both technical and user-centered aspects. Finally, we identified the core DHI-specific challenges. Based on these, a framework should guide overcoming technological limitations and offer strategies for effective communication, resource allocation, and evaluations.

### Limitations

This study has some limitations. First, because this is an emerging field, we only interviewed academic experts since they were easy to identify (through publications). Thus, our findings might not be fully transferable to cultural adaptations of DHIs in nonacademic settings. The wealth of knowledge outside of academia (eg, private sector) would add to the understanding of cultural adaptations and should be prioritized in future research. Yet, we kept our questions general and not specific to academic settings, and most of the findings are likely applicable to cultural adaptations of technology in general. Second, we used snowball sampling as a complementary recruitment method. While snowball sampling is a widely used and practical recruitment method in qualitative research, it can lead to selection bias (eg, too homogeneous sample), primarily because participants often share common professional networks, which may hinder thematic saturation. Third, culture is a multifaceted concept. This study approached culture in the context of health and technology, which is only one of many approaches to explain and understand it. Fourth, our interviewees were predominantly health and digital health experts. Including experts with a pure technology or ICT background would have likely added a valuable perspective that might be currently missing.

### Conclusions

Our work is one of the first to explore the cultural adaptation of DHIs through an academic lens. The adaptation process was described as an iterative, often unstructured, and resource-intensive process that needs careful justification and a good understanding of culture and the cultural group for which it is conducted. Based on their experiences, experts recommended that (1) the adaptation team is multiprofessional, digitally competent, and culturally sensitive (2) DHI users and (3) all other relevant stakeholders are continuously involved, and (4) that the adaptations integrate evaluations and knowledge exchange. They further emphasized that culturally adapted DHIs must be understandable, relatable, appealing, and easy to adhere to, ensuring that health technology and content reflect the target population’s lived experience, sociodemographic characteristics, and digital literacy. Yet, beyond those broader recommendations, our interviews confirmed the lack of systematic, detailed approaches to culturally adapting DHIs, including how to assess whether an adaptation is needed, how to structure the adaptation process, how to choose and prioritize adaptation elements, as well as how much to adapt to ensure that adaptations are cost-effective. That uncertainty and the added complexity that technology adds to cultural adaptations were described as critical challenges. To identify general recommendations for guiding cultural adaptations of DHIs, future research should focus on thoroughly evaluating adapted DHIs and exploring systematic ways to mitigate core challenges.

## Supplementary material

10.2196/59965 Multimedia Appendix 1 Semistructured interview guide.

## Abbreviations

DHI: digital health intervention
FRAME: Framework for Reporting Adaptations and Modifications to Evidence-based Interventions
ICT: information and communication technology
WHO: World Health Organization

## References

[1] Murray E, Hekler EB, Andersson G (2016). Evaluating digital health interventions: key questions and approaches. Am J Prev Med.

[2] Cohen AB, Mathews SC, Dorsey ER, Bates DW, Safavi K (2020). Direct-to-consumer digital health. Lancet Digital Health.

[3] Leblalta B, Kebaili H, Sim R, Lee SWH (2022). Digital health interventions for gestational diabetes mellitus: a systematic review and meta-analysis of randomised controlled trials. PLOS Digital Health.

[4] Jiang X, Ming WK, You JH (2019). The cost-effectiveness of digital health interventions on the management of cardiovascular diseases: systematic review. J Med Internet Res.

[5] Gan DZQ, McGillivray L, Han J, Christensen H, Torok M (2021). Effect of engagement with digital interventions on mental health outcomes: a systematic review and meta-analysis. Front Digital Health.

[6] Eruchalu CN, Pichardo MS, Bharadwaj M (2021). The expanding digital divide: digital health access inequities during the COVID-19 pandemic in New York City. J Urban Health.

[7] Karri K, Yarra P (2022). Inequities still exist in the use of digital health technology across different sociodemographic subgroups. Evid Based Nurs.

[8] Brewer LC, Fortuna KL, Jones C (2020). Back to the future: achieving health equity through health informatics and digital health. JMIR Mhealth Uhealth.

[9] Krukowski RA, Ross KM, Western MJ (2024). Digital health interventions for all? Examining inclusivity across all stages of the digital health intervention research process. Trials.

[10] Huh J, Koola J, Contreras A (2018). Consumer health informatics adoption among underserved populations: thinking beyond the digital divide. Yearb Med Inform.

[11] Szinay D, Forbes CC, Busse H, DeSmet A, Smit ES, König LM (2023). Is the uptake, engagement, and effectiveness of exclusively mobile interventions for the promotion of weight-related behaviors equal for all? A systematic review. Obes Rev.

[12] Western MJ, Armstrong MEG, Islam I, Morgan K, Jones UF, Kelson MJ (2021). The effectiveness of digital interventions for increasing physical activity in individuals of low socioeconomic status: a systematic review and meta-analysis. Int J Behav Nutr Phys Act.

[13] Mitchell UA, Chebli PG, Ruggiero L, Muramatsu N (2019). The digital divide in health-related technology use: the significance of race/ethnicity. Gerontologist.

[14] Gordon NP, Hornbrook MC (2016). Differences in access to and preferences for using patient portals and other ehealth technologies based on race, ethnicity, and age: a database and survey study of seniors in a large health plan. J Med Internet Res.

[15] Perski O, Short CE (2021). Acceptability of digital health interventions: embracing the complexity. Transl Behav Med.

[16] Castro FG, Barrera M, Holleran Steiker LK (2010). Issues and challenges in the design of culturally adapted evidence-based interventions. Annu Rev Clin Psychol.

[17] Nittas V, Daniore P, Chavez SJ, Wray TB (2024). Challenges in implementing cultural adaptations of digital health interventions. Commun Med (Lond).

[18] Barrera M, Castro FG, Strycker LA, Toobert DJ (2013). Cultural adaptations of behavioral health interventions: a progress report. J Consult Clin Psychol.

[19] Fendt-Newlin M, Jagannathan A, Webber M (2020). Cultural adaptation framework of social interventions in mental health: evidence-based case studies from low- and middle-income countries. Int J Soc Psychiatry.

[20] Spanhel K, Schweizer JS, Wirsching D (2019). Cultural adaptation of internet interventions for refugees: results from a user experience study in Germany. Internet Interventions.

[21] Garabiles MR, Harper Shehadeh M, Hall BJ (2019). Cultural adaptation of a scalable world health organization E-mental health program for overseas Filipino workers. JMIR Form Res.

[22] Pope C, Mays N (2019). Qualitative Research in Health Care.

[23] O’Connor C, Joffe H (2020). Intercoder reliability in qualitative research: debates and practical guidelines. Int J Qual Methods.

[24] Does my project need HRPP/IRB review?. Brown University.

[25] Marwaha JS, Kvedar JC (2021). Cultural adaptation: a framework for addressing an often-overlooked dimension of digital health accessibility. NPJ Digital Med.

[26] Stirman SW, Baumann AA, Miller CJ (2019). The FRAME: an expanded framework for reporting adaptations and modifications to evidence-based interventions. Implement Sci.

[27] Spanhel K, Balci S, Feldhahn F, Bengel J, Baumeister H, Sander LB (2021). Cultural adaptation of internet- and mobile-based interventions for mental disorders: a systematic review. NPJ Digital Med.

[28] Rodríguez MMD, Baumann AA, Schwartz AL (2011). Cultural adaptation of an evidence based intervention: from theory to practice in a Latino/a community context. Am J Community Psychol.

[29] Burchert S, Alkneme MS, Bird M (2018). User-centered app adaptation of a low-intensity e-mental health intervention for syrian refugees. Front Psychiatry.

[30] Tao D, Wang T, Wang T, Zhang T, Zhang X, Qu X (2020). A systematic review and meta-analysis of user acceptance of consumer-oriented health information technologies. Comput Hum Behav.

[31] Naderbagi A, Loblay V, Zahed IUM (2024). Cultural and contextual adaptation of digital health interventions: narrative review. J Med Internet Res.

[32] Bernal G, Bonilla J, Bellido C (1995). Ecological validity and cultural sensitivity for outcome research: issues for the cultural adaptation and development of psychosocial treatments with Hispanics. J Abnorm Child Psychol.

[33] Leong FT, Lee SH (2006). A cultural accommodation model for cross-cultural psychotherapy: Illustrated with the case of Asian Americans. Psychotherapy (Chic).

[34] Natow RS (2020). The use of triangulation in qualitative studies employing elite interviews. Qual Res.

[35] Perera C, Salamanca-Sanabria A, Caballero-Bernal J (2020). No implementation without cultural adaptation: a process for culturally adapting low-intensity psychological interventions in humanitarian settings. Confl Health.

