# Supplementary material for: Current Practice and Expert Perspectives on Cultural Adaptations of Digital Health Interventions: Qualitative Study
Source: JMIR Mhealth Uhealth. 2025 Jul 18;13:e59965. doi: 10.2196/59965 (PMC12294640; doi:10.2196/59965)
Supplement: Multimedia Appendix 1 [file mhealth-v13-e59965-s001.docx]

Preliminary Semi-structured Interview Guide

Part 1: Introduction

1. Tell me about your work related to cultural adaptations?
2. How did you define culture in the context of you work?

Part 2: Justification of cultural adaptations

1. Why did you decide to adapt [insert name of study/product]?
2. Generally, when do you think it is important to adapt a digital health product/DHI?

Part 3: Adaptation elements

1. What elements of [insert name of study/product] did you adapt?
2. How did you choose these elements?
   1. Why did you choose that approach? (----)
   2. Do you know alternative (better?) approaches?
      1. Why did you not choose one of these approaches?
3. Summarized, based on your experience, what are the most important cultural adaptation elements of a digital health product/DHI? **

Part 4: Challenges & recommendations

1. What challenges did you face during the adaptation of [insert name of study/product]?
   1. How did you overcome/solve these?
   2. What challenges do you think are unique to digital health adaptations (compared to traditional intentions)?
2. If you could go back to the adaptation of [insert name of study/product], would you do anything differently?
   1. If yes, what exactly?
3. Summarized, if you would have to provide recommendations to a fellow researcher who is about to conduct a digital health adaptation, what would that be?
